# Association between third molar agenesis and sagittal skeletal discrepancies in an orthodontic patient population

**DOI:** 10.1186/s12903-026-09539-1

**Published:** 2026-08-20

**Authors:** Philipp Schnegelsberg, Nikola Sophie Ortner, Peter Proff, Svenja Beisel-Memmert, Theodosia Bartzela, Eva Paddenberg-Schubert, Erika Calvano Küchler, Christian Kirschneck

**Affiliations:** 1 Department of Orthodontics, Medical Faculty, University Hospital Bonn, Bonn, Germany; 2 Department of Orthodontics, Faculty of Medicine, University Hospital Regensburg, University of Regensburg, Regensburg, Germany; 3 Department of Orthodontics, Carolinum Dental University Institute, Goethe University Frankfurt, Frankfurt am Main, Germany; 4 Department of Orthodontics, University Hospital Carl Gustav Carus Dresden, Technische Universität Dresden, Dresden, Germany

**Keywords:** Tooth Agenesis, Third molars, Skeletal Class III, Tooth agenesis pattern, Orthodontic patients

## Abstract

**Background:**

Previous studies have reported differences in the prevalence of tooth agenesis according to sagittal skeletal discrepancies, while third molars have often not been considered. The aim of the present study was to investigate the association between third molar agenesis and sagittal skeletal discrepancies.

**Methods:**

This cross-sectional study comprised 484 orthodontic patients. Pre-orthodontic lateral cephalometric radiographs were digitally analyzed using OnyxCeph³. SNA, SNB, and ANB angles were determined, and the skeletal classification based on ANB was performed as Class I, Class II, and Class III. In addition, sagittal jaw positions were classified as retrognathic, orthognathic, or prognathic. Third molar agenesis was evaluated on panoramic radiographs. The control group consisted exclusively of individuals with a complete permanent dentition of 32 teeth. Statistical analysis was performed at α = 0.05.

**Results:**

A total 244 females and 240 males were included: 216 patients had skeletal Class I malocclusion, 201 had skeletal Class II malocclusion, and 67 had skeletal Class III malocclusion. A total of 117 patients had third molar agenesis. Patients with skeletal Class III malocclusion showed a higher prevalence of third molar agenesis (total) (*p* = 0.0086) and a higher prevalence of maxillary third molar agenesis (*p* = 0.0003). The number of missing third molars ranged from 1 to 4, and a statistically significant difference was observed, with patients with skeletal Class III malocclusion showing a higher number of missing third molars compared with patients with skeletal Class II malocclusion (*p* = 0.0309) and skeletal Class I malocclusion (*p* = 0.0432). Logistic regression analysis was performed, in which gender and age were included in the model as covariates showed that Skeletal class III malocclusion remained associated with third molar agenesis (*p* < 0.05).

**Conclusion:**

Third molar agenesis was significantly more prevalent in patients with skeletal Class III malocclusion.

## Background

Tooth agenesis, defined as the congenital absence of one or more primary or permanent teeth, represents the most prevalent developmental alteration of the human craniofacial complex [1, 2]. It occurs more frequently in the permanent dentition and most commonly affects the third molars [3]. The global prevalence of third molar agenesis has been reported at approximately 22.6%, although some variability exists between studies and populations [4]. Among all teeth, third molars demonstrate considerable developmental variability [4, 5]. As the final teeth to initiate and complete development, they exhibit instability in timing and morphogenesis, rendering them particularly prone to agenesis and other developmental dental alterations [5, 6].

Sagittal skeletal discrepancies are commonly classified according to the anteroposterior relationship between the maxilla and mandible and represent a fundamental component of orthodontic diagnosis and treatment planning. Skeletal Class I describes a harmonious sagittal jaw relationship, whereas skeletal Class II is characterized by a distal sagittal relationship between the mandible and maxilla, and skeletal Class III by a mesial sagittal relationship. These discrepancies may result from maxillary, mandibular, or combined skeletal variations and are typically evaluated using cephalometric parameters [7].

Associations between tooth agenesis and craniofacial morphology have been described in orthodontic populations [8, 9]. Furthermore, third molar agenesis has been linked to craniofacial size and shape variation in geometric morphometric studies [10–12]. Despite increasing evidence linking tooth agenesis to craniofacial morphology, the association between third molar agenesis and sagittal skeletal discrepancies remains insufficiently clarified. Therefore, the aim of the present study was to examine the association between sagittal skeletal patterns and third molar agenesis in a group of German orthodontic patients.

## Methods

### Design and study setting

This cross-sectional study was conducted to investigate the prevalence and patterns of third molar agenesis and its possible association with sagittal skeletal patterns in a group of German orthodontic patients. The reporting of this study followed the STROBE (Strengthening the Reporting of Observational Studies in Epidemiology) guidelines [13]. The study protocol was approved by the Ethics Committee of the University Hospital Bonn and the University Hospital Regensburg, Germany (approval number: 2024-252-BO and 19-1549-101).

Patient records and radiographs obtained as part of routine orthodontic diagnostics between 2020 and 2025 were retrospectively reviewed.

### Sample characteristics

Panoramic radiographs were used as the primary diagnostic tool because they provide comprehensive visualization of the entire dentition in all four quadrants and allow reliable assessment of third molar development. Each radiograph was systematically examined for the presence of tooth germs, crowns, or other detectable developmental stages of the third molars. If no radiographic evidence of third molar formation was present within the anatomically expected region, the tooth was classified as congenitally absent.

### Study selection and data collection

To ensure diagnostic validity and to minimize misclassification due to ongoing dental development, only patients aged 14 years or older were included, as third molar mineralization is usually sufficiently advanced at this age [6]. All radiographic assessments were performed under standardized conditions, with careful evaluation of image quality and anatomical landmarks, in order to maintain consistency and reproducibility within the study sample. For the case and control groups, patients with syndromic conditions, cleft lip and/or palate, teeth extraction, previous orthodontic treatment, facial trauma, facial surgery, or unclear documentation regarding previous extraction of third molars were excluded to reduce potential bias and ensure data integrity.

For statistical analysis, the study population was divided into two groups. The study group consisted of patients presenting with agenesis of at least one third molar, whereas the control group included individuals with a complete permanent dentition of 32 teeth (none of the cases have tooth agenesis or dental extraction).

The sample size was determined with reference to previously published studies investigating the association between craniofacial patterns and third molar agenesis in orthodontic patients [8]. Based on a frequency difference of 10% (α = 0.05, β = 0.80), the calculated sample size was approximately 480 individuals. Participants were included consecutively until the required sample size was reached.

For evaluation of sagittal skeletal patterns, pre-orthodontic lateral cephalometric radiographs were digitally analyzed using OnyxCeph³. SNA, SNB, and ANB angles were determined using the reference points Sella, Nasion, Point A, and Point B. Sagittal skeletal discrepancies were assessed based on the anteroposterior relationship between the maxilla and mandible, which represents a fundamental component of orthodontic diagnosis and treatment planning. Skeletal Class I was defined as ANB = 0°–4°, skeletal Class II as ANB > 4°, and skeletal Class III as ANB < 0°. Skeletal Class I indicates a harmonious sagittal jaw relationship, whereas skeletal Class II is characterized by a distal sagittal relationship between the mandible and maxilla, and skeletal Class III by a mesial sagittal relationship. These discrepancies may result from maxillary, mandibular, or combined skeletal variations. Sagittal relationships were evaluated using cephalometric parameters, most commonly the ANB angle, which reflects the relative position of the maxilla and mandible in relation to the cranial base [7].

### Statistical analysis

All statistical analyses were carried out using GraphPad Prism for Windows, version 11.0.0 or IBM SPSS Statistics for Windows, Version 31.0 (IBM Corp., Armonk, NY, USA). Differences in the prevalence of third molar agenesis between groups were assessed using the chi-square test for categorical variables. Before comparing the number of missing third molars between groups, the distribution of the data was tested for normality using the Shapiro–Wilk test. As the data did not follow a normal distribution, group comparisons were performed using the non-parametric Mann–Whitney U test.

Multinomial logistic regression analysis was performed to evaluate the association between third molar agenesis (independent variable) and the type of skeletal malocclusion (dependent variable: Class I, Class II, and Class III).

To investigate whether third molar agenesis severity (number of missing teeth) is associated with skeletal malocclusion patterns, a multinomial logistic regression model was also fitted. Gender and age were included in the model as covariates to adjust for potential confounding effects.

All tests were two-sided, and the significance level was set at α = 0.05.

Possible confounding effects were reduced by applying predefined inclusion and exclusion criteria, thereby limiting heterogeneity within the study population. Additional multivariable adjustment was not performed, since the main analyses were based on predefined group comparisons.

No missing data were present in the final dataset, as records with incomplete or non-evaluable information had been excluded before statistical analysis, resulting in a complete-case analysis, therefore no imputation methods were used.

## Results

After inclusion and exclusion criteria, a total of 484 patients were included: 244 (50.4%) females and 240 (49.6%) males. The age ranged from 13 to 36 years old (mean age = 13.6, standard deviation = 1.7). Two hundred sixteen (44.6%) patients had skeletal Class I malocclusion, 201 (41.5%) had skeletal Class II malocclusion, and 67 (13.8%) had skeletal Class III malocclusion. 117 (24.2%) patients had third molar agenesis (at least one third molar was congenitally missing). Skeletal malocclusion and third molar agenesis were not statistically different between sexes, as presented in Table 1.

**Table 1:** Prevalence of third molar agenesis by sex, sample characteristics, and subtypes

Variables	*N*
*Sex distribution*	
Female	244
Male	240
*Third molar agenesis characteristics*	
Third molar agenesis (at least one third molar missing)	117
Maxillary third molar agenesis	77
Mandibular third molar agenesis	73
Third molar agenesis associated with another missing tooth/teeth	12
Unilateral third molar agenesis	51
Bilateral third molar agenesis	66

Table 2 presents third molar agenesis according to skeletal malocclusion. Patients with skeletal Class III malocclusion had a higher prevalence of third molar agenesis (total) (*p* = 0.0086) and a higher prevalence of maxillary third molar agenesis (*p* = 0.0003).

**Table 2:** Third molar agenesis according to skeletal malocclusion classes I, II, and III

Groups	Skeletal Class I	Skeletal Class II	Skeletal Class III	*p*-value
No Agenesis (Controls)	166 (45.2)	160 (43.6)	41 (11.2)	reference
Third molar agenesis (total)	50 (42.7)	41 (35.0)	26 (22.2)	**0.0086**
Maxillary third molar agenesis	30 (38.9)	25 (32.5)	22 (28.6)	**0.0003**
Mandibular third molar agenesis	32 (43.8)	29 (39.7)	12 (16.4)	0.4402

Note: All comparisons were performed using individuals with complete dentition (32 teeth) as the control group. The chi-square test was applied. Bold values indicate statistically significant differences

Table 3 presents the third molar agenesis according to the maxillary phenotypes. The presence of third molar agenesis was not statistically different among patients with orthognathic, retrognathic and prognathic maxilla (*p* > 0.05).

**Table 3:** Third molar agenesis according to the maxillary position

Groups	Orthognathic	Retrognathic	Prognathic	*p*-value	
No Agenesis (Controls)	132 (36.0)	156 (42.5)	79 (21.5)	reference	
Third molar agenesis (total)	51 (43.6)	46 (39.3)	20 (17.1)	0.2965	
Maxillary third molar agenesis	37 (48.0)	29 (37.7)	11 (14.3)	0.1083	
Mandibular third molar agenesis	30 (31.7)	29 (46.1)	14 (22.2)	0.8032	

Note: All comparisons were performed using individuals with complete dentition (32 teeth) as the control group. The chi-square test was applied

Table 4 presents the third molar agenesis according to the mandible phenotypes. The presence of third molar agenesis was not statistically different between patients with orthognathic, retrognathic, and prognathic mandible (*p* > 0.05).

**Table 4:** Third molar agenesis according to the mandibular position

Groups	Orthognathic	Retrognathic	Prognathic	*p*-value	
No Agenesis (Controls)	112 (28.6)	207 (58.0)	48 (13.4)	reference	
Third molar agenesis (total)	35 (29.9)	60 (51.3)	22 (18.8)	0.2908	
Maxillary third molar agenesis	25 (32.5)	38 (49.3)	14 (18.2)	0.3397	
Mandibular third molar agenesis	21 (28.8)	39 (53.4)	13 (17.8)	0.5963	

Note: All comparisons were performed using individuals with complete dentition (32 teeth) as the control group. The chi-square test was applied

The number of missing third molars ranged from 1 to 4. Fifty patients (10.3%) had one missing third molar, 35 patients (7.2%) had two missing third molars, 12 patients (2.5%) had three missing third molars, and 20 patients (4.1%) had four missing third molars.

**Fig. 1:**
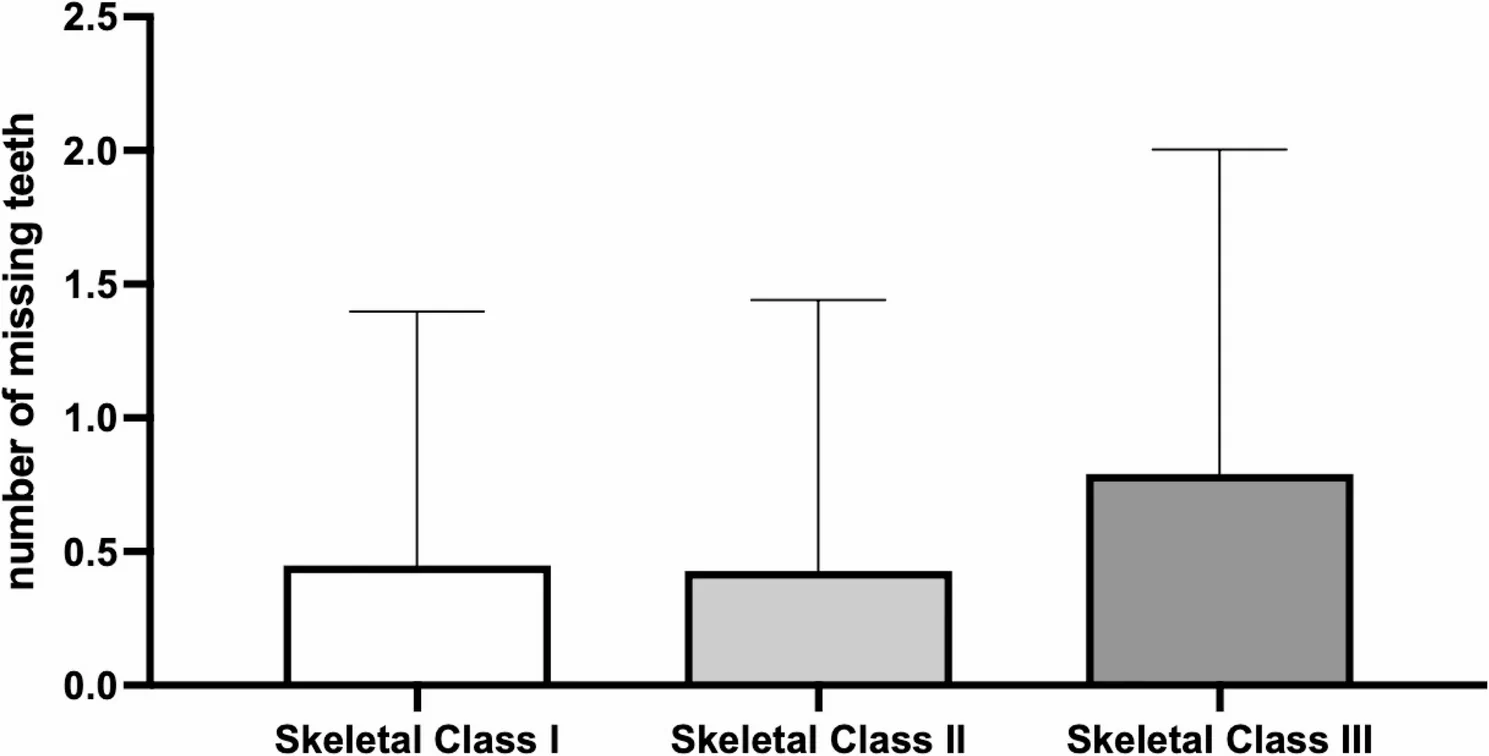
Mean number of congenitally missing third molars by skeletal malocclusion, statistical comparison performed using the Mann–Whitney U test

The mean number of missing third molars was 0.44 (standard deviation 0.94) in patients with skeletal Class I, 0.42 (standard deviation 1.01) in patients with skeletal Class II, and 0.79 (standard deviation 1.21) in patients with skeletal Class III, as presented in Fig. 1. A statistically significant difference was observed. Patients with skeletal Class III malocclusion showed a higher number of missing third molars compared with patients with skeletal Class II malocclusion (*p* = 0.0309) and skeletal Class I malocclusion (*p* = 0.0432).

Logistic regression analysis was performed, in which gender and age were included in the model as covariates to adjust for potential confounding effects. These results are presented in the Table 5. Skeletal class III malocclusion remained associated with third molar agenesis (*p* < 0.05).

**Table 5:** Logistic regression analysis to analyze the presence of third molar agenesis

Skeletal Malocclusion	Variable	B	Standard Error	Exp(B)	95% CI for Exp(B)		*p*-value
					Lower Bound	Upper Bound	
Class II	Age	-0.10	0.06	0.90	0.78	1.03	0.129
	Gender*	0.27	0.19	1.31	0.89	1.93	0.167
	Without third molar agenesis	0.11	0.24	1.12	0.69	1.79	0.644
Class III	Age	-0.05	0.08	0.94	0.80	1.11	0.532
	Gender	-0.35	0.28	0.70	0.40	1.23	0.222
	Without third molar agenesis	-0.78	0.30	0.45	0.25	0.82	**0.010**

Note: Skeletal Class I was the reference

Bold values indicate statistically significant differences

*Male was the reference

The logistic regression model to investigate the severity of third molar agenesis (number of missing teeth) according to the skeletal malocclusion, gender and age were included in the model as covariates to adjust for potential confounding effects, is presented in the Table 6. Skeletal class III malocclusion remained associated with the number of missing third molars (*p* < 0.05).

**Table 6:** Logistic regression analysis to analyze the severity of third molar agenesis according to the skeletal malocclusion

Skeletal Malocclusion	Variable	B	Standard Error	Exp (B)	95% CI for Exp (B)		*p*-value
					Lower Bound	Upper Bound	
Class II	Age	-0.11	0.07	0.89	0.78	1.03	0.177
	Gender*	0.27	0.19	1.31	0.89	1.93	0.169
	Number of missing third molars	0.01	0.10	1.0	0.82	1.24	0.934
Class III	Age	-0.06	0.08	0.93	0.79	1.10	0.425
	Gender	-0.37	0.28	0.68	0.39	1.20	0.192
	Number of missing third molars	0.304	0.12	1.35	1.1	1.72	**0.014**

Note: Skeletal Class I was the reference

Bold values indicate statistically significant differences

*Male was the reference

## Discussion

The relationship between nonsyndromic tooth agenesis and craniofacial morphology has been investigated extensively across diverse populations [8–12]. Tooth agenesis represents the most prevalent developmental anomaly of the human dentition [1, 2], and among permanent teeth, third molars are most frequently affected [3, 4]. Owing to their late initiation and prolonged development, third molars exhibit considerable biological variability, which may partly explain their high susceptibility to agenesis [5, 6]. A systematic review with meta-analysis [4] demonstrated that the prevalence of third molar agenesis varies considerably between populations, with an overall worldwide prevalence of approximately 22.6% and values ranging from about 5% to more than 50% depending on the geographic region. In European populations, the reported prevalence is approximately 21–22%. The prevalence of 24% observed in the present study is therefore consistent with previous reports.

Beyond isolated dental findings, several studies have suggested that reduced tooth number due to developmental agenesis may be associated with craniofacial size and shape. Geometric morphometric analyses have shown that individuals with fewer teeth tend to exhibit smaller craniofacial dimensions and altered facial morphology [10–12]. In particular, third molar agenesis has been associated with reduced facial size and changes in craniofacial configuration [12].

In the present study, a significantly higher prevalence of third molar agenesis was found in individuals with skeletal Class III compared with individuals with skeletal Class I and Class II. This difference was particularly evident for maxillary third molar agenesis, whereas no significant differences were detected when patients were classified according to isolated maxillary or mandibular position based on SNA and SNB angles. These findings further support the concept that dental development and craniofacial growth are biologically interconnected processes.

Previous investigations have described associations between tooth agenesis and reduced cranial base length, shorter maxillary dimensions, and skeletal Class III configurations [4, 9, 12]. In the sagittal dimension, an increased prevalence of third molar agenesis has been reported in individuals presenting with skeletal Class III patterns [8]. In agreement with these reports, the present study also demonstrated that patients with skeletal Class III showed a significantly higher overall prevalence of third molar agenesis compared with skeletal Class I and Class II. In addition, maxillary third molar agenesis occurred more frequently in the Class III group, whereas no significant differences were found when sagittal jaw position was analyzed separately using SNA and SNB angles.

The biological mechanisms underlying this association remain incompletely understood. One possible explanation relates to shared genetic pathways influencing both odontogenesis and craniofacial skeletal development. Previous literature has suggested that tooth agenesis may reflect broader developmental variation within the craniofacial complex rather than representing an isolated dental anomaly [1, 2]. This skeletal tooth interaction is likely mediated by shared genetic pathways, such as GLI2-related, exerting a stronger influence on third molar development than on other molars [14]. From an evolutionary perspective, third molar agenesis has also been discussed as part of a long-term reduction in tooth number associated with changes in craniofacial size in modern humans [5].

In contrast to three-dimensional geometric morphometric investigations that have demonstrated subtle associations between tooth number and overall craniofacial shape [10–12], the present study did not detect significant associations between third molar agenesis and isolated linear or angular cephalometric parameters. This divergence likely reflects methodological differences. Three-dimensional morphometric techniques quantify global shape variation across the entire craniofacial complex. In contrast, conventional two-dimensional cephalometric analysis is restricted to predefined angular and linear measurements and may therefore lack sensitivity for detecting subtle, spatially distributed morphological variation. Moreover, transverse skeletal relationships were not assessed.

It is noteworthy that several previous studies reporting statistically significant associations described mean values that remained within normative ranges [4, 9, 12], indicating that the observed differences were modest in magnitude. Accordingly, small effect sizes in the present cohort may not have reached statistical significance, particularly in subgroup analyses with limited sample sizes.

Skeletal classification in this study was based on the conventional ANB angle. Although widely used in orthodontic diagnostics, the ANB angle is influenced by vertical jaw relationships and cranial base morphology, which may affect its capacity to represent true sagittal discrepancies [15–17]. This may have affected our data, as skeletal Class III patients frequently exhibit a shorter cranial base and a steeper mandibular plane, which can result in artificially reduced ANB values. Consequently, some cases may have been misclassified as Class I, potentially underestimating the true association [18]. The observed association between third molar agenesis and skeletal Class III should therefore be interpreted within the constraints of this diagnostic framework. Future investigations incorporating individualized sagittal assessments, alternative indicators as previously reported [19] such as the individualized ANB angle described by Paddenberg et al. [20], or three-dimensional imaging, may help clarify whether the association is dependent on the classification method applied [21]. Variants in genes such as PAX9, typically produce symmetric patterns, while unilateral cases more likely reflect variable expression or local factors. Previous reports have suggested a higher prevalence of bilateral maxillary third molar agenesis in Class III individuals in specific populations. Stratifying agenesis laterality by skeletal class in future studies may clarify whether bilateral patterns are more common in Class III individuals, and support shared odontogenic-craniofacial pathways [22].

Several limitations must be acknowledged. The retrospective cross-sectional design precludes causal inference. The study population consisted exclusively of orthodontic patients treated in universities-based setting, which may limit generalizability. This subgroup of population is more likely to present skeletal Class III malocclusion and dental anomalies and might not represent the general population. As the cohort primarily comprised adolescents, residual craniofacial growth after radiographic assessment may have altered sagittal relationships during subsequent development. Another potential limitation that should be highlighted is the lack of adjustment for multiple comparisons introduces a potential risk of Type I error, potentially leading to false-positive associations between third molar agenesis and skeletal malocclusions. In addition, the relatively small number of skeletal Class III patients may have reduced statistical power in subgroup analyses.

Despite these limitations, the study presents notable strengths. The analysis was conducted in a well-defined contemporary orthodontic cohort with clearly specified inclusion and exclusion criteria. Age restrictions minimized the risk of misclassifying late third molar mineralization as agenesis, radiographic assessment was performed systematically, and skeletal classification followed predefined cephalometric criteria, ensuring consistent group allocation.

Over time, third molars have been systematically excluded from agenesis research as part of established methodological conventions, particularly in pattern-based analyses such as the Tooth Agenesis Code [23]. While this approach ensured comparability across agenesis patterns and craniofacial anomaly studies, it also omitted the third molars, a tooth type that is highly informative for agenesis patterns and their links to craniofacial morphology. As the terminal element of the molar developmental field and particularly sensitive to odontogenic disturbances, third molars show the greatest developmental variability and increased susceptibility to agenesis [24]. The systematic exclusion of third molars represents an important methodological omission in the existing literature. Future agenesis pattern studies should reconsider the routine exclusion of third molars, particularly when investigating craniofacial morphological correlates.

From a clinical perspective, the observed association between skeletal Class III configuration and an increased prevalence of third molar agenesis suggests that third molar agenesis may represent a phenotypic marker of sagittal skeletal discrepancy. Recognition of this relationship may support early and targeted radiographic evaluation in patients presenting with Class III patterns and may contribute to integrated orthodontic treatment planning that considers both skeletal morphology and dental development. Incorporating craniofacial pattern analysis into the assessment of third molar agenesis could therefore enhance diagnostic precision and risk stratification in orthodontic patients.

In routine orthodontic practice, third molar agenesis may serve as a relevant anatomical and biomechanical consideration when planning treatment for patients with skeletal Class III malocclusion. Clinically, the congenital absence of mandibular third molars may facilitate lower arch distalization by reducing dental constraints in the retromolar space, potentially supporting non-extraction treatment mechanics and reducing treatment complexity in selected cases. Conversely, missing maxillary third molars may influence the available posterior dental anatomy during maxillary protraction or alignment. For severe Class III cases requiring orthognathic surgery, mandibular third molar agenesis may simplify bilateral sagittal split osteotomies and potentially reduce the risk of unfavorable fractures associated with impacted third molars or their roots in the osteotomy region. Therefore, assessment of third molar status may contribute to individualized treatment planning, particularly when evaluating the feasibility of orthodontic camouflage, the available posterior space, and the need for combined orthodontic and surgical treatment [25]. Overall, the present findings reinforce the concept that third molar agenesis is not merely an isolated dental anomaly but may be linked to broader craniofacial developmental patterns.

## Conclusion

Third molar agenesis was significantly more prevalent in patients with skeletal Class III compared with skeletal Class I and Class II individuals in this orthodontic cohort. No significant association was observed between the isolated sagittal positions of the maxilla or mandible (SNA, SNB) and third molar agenesis.

## Data Availability

The datasets generated and analyzed during the current study are not publicly available because they contain potentially identifiable patient information and are subject to ethical and data protection restrictions. Data are available from the corresponding author upon request.
